# Hiring Discrimination in the Organisational Context: A Harmonized Factorial Survey Dataset from Four European Countries (Germany, Norway, Poland, Romania)

**DOI:** 10.1038/s41597-026-07187-2

**Published:** 2026-04-17

**Authors:** Dominik Buttler, Christian Imdorf, Vegar Bjørnshagen, Anatolie Cosciug, Katarzyna Lipowska, Iga Magda, Marta Palczyńska, Mateusz Smoter, Sara Ayllón, Justyna Bell, Mateusz Krząkała, Iuliana Precupetu, Rosa Maria Radogna, Jon Rogstad, Robin Samuel, Laura Tufa, Ona Valls, Nick Wessel

**Affiliations:** 1 Department of Labour and Social Policy, Poznań University of Economics and Business, Poznań, Poland; 2 Institute of Sociology, Leibniz University Hannover, Hannover, Germany; 3 OsloMet – Oslo Metropolitan University, NOVA, Oslo, Norway; 4 West University of Timişoara, Timişoara, Romania; 5 Institute for Structural Research, Warsaw, Poland; 6 SGH Warsaw School of Economics, Warsaw, Poland; 7 Faculty of Economic Sciences, University of Warsaw, Warsaw, Poland; 8 Department of Economics, University of Girona, Girona, Spain; 9 Research Institute of the University of Bucharest, University of Bucharest, Bucharest, Romania; 10 Research Institute for Quality of Life, Romanian Academy, Bucharest, Romania; 11 Faculty of Sociology and Social Work, University of Bucharest, Bucharest, Romania; 12 Department of Social Sciences, University of Luxembourg, Esch-sur-Alzette, Luxembourg; 13 Department of Research Methods and Diagnosis in Education, University of Barcelona, Barcelona, Spain

## Abstract

Hiring discrimination has been extensively documented in numerous studies. It is not driven solely by individual actions but is context-dependent, shaped – among other factors – by organisational features that enable or constrain discriminatory behaviour. However, little is known about why some organisations, such as companies or public agencies, discriminate more than others. This data descriptor presents the dataset from a harmonised factorial survey experiment (FSE) on hiring discrimination, which enabled detailed analyses of discriminatory behaviour and the organisational contexts that shape it. The FSE measured discrimination based on nationality, gender and care obligations and was conducted between 2024 and 2025 in four European countries—Germany, Norway, Poland and Romania—among 2,506 recruiters. The dataset includes 15,036 evaluations of hypothetical job candidates for medium-skilled positions, complemented by extensive information on recruiters and the organisations they represent.

## Background & Summary

Discriminatory hiring practices are a major barrier to employment for vulnerable groups based on characteristics such as nationality, gender, age, disability and care obligations. This phenomenon has been extensively studied using correspondence tests, where researchers submitted fictitious applications to real job postings^1–5^. These applications featured nearly identical candidates who differed only in specific characteristics being tested for discrimination. However, hiring discrimination is not solely an individual phenomenon but is context-dependent and moderated by organisational characteristics, among other factors, that enable or constrain discriminatory behaviour. These factors create the so-called ‘opportunity structure’ for discrimination^6^.

In correspondence tests, it is difficult to collect in-depth information about participating organisations without revealing a study’s purpose. Such organisational data are more often obtained from observational studies that rely on questionnaires or qualitative interviews. However, these sources often do not allow for a convincing identification of discriminatory behaviour. The research project PATHS2INCLUDE^7^ that produced the ‘Hiring Discrimination in the Organisational Context’ dataset addressed this gap via a cross-nationally harmonised factorial survey experiment (FSE) on hiring intentions embedded in a comprehensive questionnaire about organisational features. Therefore, it combined the rigour of an experimental design with detailed organisational data. The survey was conducted in 2024–2025 among 2,506 recruiters in four European countries: Germany, Norway, Poland and Romania. Each recruiter evaluated the hiring likelihood of six fictitious candidates (15,036 in total), assuming they had applied for a medium-skilled job in their organisation (i.e., information and communication technology (ICT) technician, bookkeeping clerk, office clerk, secretary or sales worker).

The FSE focused on three types of discrimination with respect to candidates’ nationality, care obligations and gender. The first criterion is highly relevant today due to recent increases in migration flows driven by conflicts and economic instability in regions such as the Middle East or Africa, as well as by the war in Ukraine, which has significantly increased migration into many European countries. In the FSE, we compared the hiring chances of three national groups: natives, Ukrainians and a third group representing migrants who were perceived as more culturally or socially distant from the native populations (i.e., Syrians in Germany and Norway, Nepalis in Romania and Belarusians in Poland). In this experiment, we examined the role of nationality as well as other barriers to immigrants’ employment, including language skills, access to referral networks, and the transferability of foreign qualifications. Care responsibilities shape gendered inequalities in the labour market, as shown by the divergence in men’s and women’s career trajectories and wages after becoming parents. The importance of this phenomenon was recently highlighted in the social sciences by the Nobel Memorial Prize in Economic Sciences, awarded to Claudia Goldin in 2023.

In this FSE, care responsibilities were represented by two variables: partnership status and parenthood status. This novel approach allowed for more flexible modelling, as previous experimental research on care-based discrimination focused mainly on parental status and did not examine its interaction with partnership status completely or consistently. The FSE controlled for gender, which can itself be a basis for discrimination and may interact with nationality and care obligations in shaping hiring intentions. The vignettes presented to recruiters also included other candidate attributes typically available at the early stages of the recruitment process.

Norway, Germany, Poland, and Romania were selected to capture meaningful cross-country variation in welfare regimes, family policies, gender norms, and immigrant integration frameworks that were expected to shape hiring discrimination. These institutional and normative contexts influence employers’ incentives and constraints, thereby affecting discrimination with respect to gender, nationality, and care obligations.

To summarise, the publicly available raw dataset from the survey allows for detailed analyses of, inter alia:

- Hiring discrimination related to nationality, care obligations, gender and their interactions
- Organisational features that may moderate hiring discrimination, including the design and formalisation of recruitment and selection processes, characteristics of a recruitment board, opportunities for professional development, flexible working arrangements, diversity-oriented aims and measures, an organisation’s gender and ethnic composition, company size, internationalisation, location, sector of activity and ownership type (public or private)
- Job characteristics that may moderate hiring discrimination, such as the type of job being applied for, the level of interaction with customers and co-workers and job time demands
- Recruiter characteristics that may moderate hiring discrimination, including education level and field, professional experience and sociodemographic attributes
- Cross-country comparisons of hiring discrimination

## Methods

### Ethics

Institutions responsible for this study were members of the consortium of the research project Paths2Include supported by the European Union’s Horizon Europe Research and Innovation Programme (Grant Agreement No. 101094626):

- Oslo Metropolitan University (OsloMet), Norway (project leader)
- Leibniz University Hannover (LUH), Germany
- Institute for Structural Research (IBS), Poland
- Research Institute of the University of Bucharest (ICUB), Romania

Based on an evaluation by the Norwegian Agency for Shared Services in Education and Research (SIKT), no ethics approval was deemed necessary, and GDPR Article 6(1)(a) and Article 9(2)(a) provided a sufficient legal basis for the processing of personal data. Therefore, arranging ethics committee approvals was left to the discretion of research consortium members, who obtained approval from their respective local ethics bodies where required. The following actions were undertaken by research teams involved in the survey:

- OsloMet: evaluation of SIKT (see above)
- LUH: according to the Research Officer in the Presidential Staff there was no obligation at Leibniz University Hannover to obtain an ethics vote
- IBS: the team registered the survey with the German Association for Experimental Economic Research e.V. and was granted the Institutional Review Board Certificate (ethics certificate) No. uJk64HDA, https://gfew.de/ethik/uJk64HDA
- ICUB: the team received approval of the Ethics Committee of the University of Bucharest, registration number 62/9.05.2024

All survey activities were conducted in accordance with relevant guidelines and regulations. The online version of the survey was implemented by GfK Polonia, which used subcontracted respondent panels, i.e. pre-recruited samples of individuals who had agreed to participate in research studies conducted by the survey company managing the panel. The study was anonymous, and no personal data were collected as part of it. The survey questionnaire also did not include any open-ended questions (which could theoretically have enabled the identification of respondents). Detailed information regarding the privacy policy was presented to respondents prior to their participation in the study, which was voluntary in nature. In Germany, where additional data –names and business email addresses—were collected to send survey invitations (see “Sampling” section), respondents were informed of their GDPR rights, including the right of access, rectification, erasure, and the right to object to data processing. The collection of these data and the use of contact information were carried out in accordance with the GDPR. All participants provided informed consent prior to the data collection.

### Factorial survey experiment as a survey method

To investigate hiring discrimination, we conducted an FSE in which experienced recruiters from Germany, Norway, Poland and Romania evaluated hypothetical job candidates described in vignettes (i.e., standardised descriptions of fictitious candidates applying for jobs). The vignettes differed with respect to some characteristics (dimensions) whose values (levels) varied randomly.

Properly designed FSEs feature orthogonal (uncorrelated) vignette dimensions, which enhances statistical efficiency^8^. The method also allows full control of the presented information, thereby reducing bias from unobserved variables. Critics argue that FSEs measure intentions rather than actual behaviour, suggesting that hiring discrimination is better identified by correspondence tests in which fictional applications are sent to real job advertisements and actual recruiter responses are measured. A recent study reported discrepancies between the two methods^9^. However, some of its claims have been questioned due to potential methodological limitations^10^, and there is evidence showing consistency of FSEs with field experiments^11,12^. We chose FSEs over correspondence tests because the latter, while effective for studying hiring behaviour, cannot easily be combined with surveys to capture organisational-level factors.

### Vignette dimensions and design

The range of dimensions presented to the respondents in vignettes was critically determined based on a literature review^13–25^ and limited to what is usually available to recruiters in the early recruitment stages. The exception was partnership and parenthood status, as caring obligations were an important criterion of discrimination in the research project. This information is not usually requested during an interview, but it was assumed that such details would be relatively easy to observe. Moreover, in four analysed countries, it is not uncommon for candidates to disclose parenthood and partnership status at an early stage of the recruitment process. The dimensions included in the vignettes and their levels are shown in Table 1.

**Table 1:** FSE. Dimensions and their levels used in the vignettes.

Dimensions	Levels
Dim1: referrals	1. Received the application directly from a candidate 2. Candidate was recommended by an employee
Dim2: gender	1. Woman 2. Man
Dim3: nationality	1. Host country [Germany/Norway/Poland/Romania] 2. Host country [Germany/Norway/Poland/Romania] 3. Culturally closer country [Ukraine] 4. Culturally more distant country [Syria/Belarus/Nepal]
Dim4: country where graduated	1. Host country 2. Home country
Dim5: level of host-country language	1. Proficient level (C2) 2. Upper-intermediate level (B2)
Dim6: partnership status	1. Candidate lives alone 2. Candidate lives with a partner/spouse
Dim7: parenthood status	1. Candidate raises a preschool-aged child 2. Candidate has no children
Dim8: type of work experience	1. Two years in the host country in a similar position 2. Two years in the host country, not related to the applied-for job

Note: To approximate the actual applicant pools and to increase psychological realism, the number of native applicants in Dim3 was doubled. Technically, this was achieved by assigning two values to the ‘origin country’ level. The practical disadvantage of this solution was that a pair of vignettes in one deck (out of 24) was identical. The respondents were informed of this possibility.

Drawing on the theory of cultural distance that might moderate hiring discrimination^26^, one culturally closer and one more distant national group were selected for each country in *Dim3*. For policy-relevant reasons, immigrants from Ukraine were chosen as the culturally closer group in all four countries, as their numbers rose sharply after Russia’s full-scale invasion in 2022. For Norway and Germany, the culturally distant groups were immigrants from Syria, while for Romania they were from Nepal—the largest non-European groups in these countries. For Poland, Belarusians were selected as the second national group as an exception, despite their small cultural distance from the Poles. The case of Belarusians was considered relevant because they constituted the second-largest minority, and public attitudes towards them were more negative than towards Ukrainians due to the political situation at the time of the study.

To better understand the mechanisms of ethnic discrimination, the vignettes incorporated dimensions correlated with both nationality and hiring outcomes: language skills^27^ proxied by *Dim5*, limited transferability of foreign qualifications^23,24^ (*Dim4)* and restricted access to referral networks^28^ (*Dim1*). Also, all candidates were assigned two years of host-country experience, signalling at least partial integration into the labour market and society (*Dim8*).

The proxies for caregiving responsibilities can be derived from two vignette dimensions: partnership status (*Dim6*) and parenthood status (*Dim7*). This is a novel approach that offers greater flexibility in modelling, as previous experimental research on care-based discrimination has focused mainly on parental status, while its interaction with partnership status has not been addressed in a complete or unified way^29^. The gender of candidates (*Dim2*) can be analysed as a separate factor affecting hiring chances, in interaction with nationality or care dimensions, or in relation to the specific jobs for which the hypothetical candidates applied.

The vignette design allowed for 512 potential combinations of candidate characteristics (all combinations of dimension levels presented in the Table 1, i.e., 2^7 × 4). However, two implausible cases were ruled out by design – native candidates with low proficiency in their native language (Dim3 <  = 2 & Dim5 = 2) and native candidates holding educational credentials from immigrant-origin countries (Dim3 <  = 2 & Dim4 =2). To handle the large design, we used the SAS %Mktex macro^8,30^, which selects a smaller, statistically efficient subset of vignettes while maintaining key design properties such as orthogonality and balance and enforcing predefined design constraints arising from implausible combinations.

We implemented a Resolution IV design, enabling the estimation of all main effects and all two-way interactions except for the two variables involved in the excluded combinations. This process produced 144 vignettes with a D-efficiency of 86.46%, after which further increases in the vignette sample yielded minimal gains. The selected vignettes were grouped into 24 decks (each containing six vignettes) and randomly assigned to respondents. The order of vignettes within decks was also randomized. Table 2 shows that the dimensions were uncorrelated with each other, as well as with deck numbers and vignette order within decks, confirming that the randomization process was efficient. Only Dim3, Dim4, and Dim5 were correlated with each other, which is an effect of excluding two implausible combinations. In estimating the determinants of hiring, the correlation between these independent variables may reduce statistical efficiency by inflating the standard errors of the estimated main effects. However, this is unlikely to substantially affect the results given the moderate correlation and the relatively large sample size.

**Table 2:** Pairwise Correlations among Dimensions.

Dimensions	Dim1	Dim2	Dim3	Dim4	Dim5	Dim6	Dim7	Dim8	Vorder	Deck
Dim1: referrals	1.000									
Dim2: gender	0.006	1.000								
Dim3: nationality	0.007	0.006	1.000							
Dim4: country where graduated	−0.008	−0.008	0.432	1.000						
Dim5: level of host country language	0.007	0.016	0.430	0.247	1.000					
Dim6: partnership status	0.004	0.009	−0.005	0.002	0.001	1.000				
Dim7: parenthood status	0.001	−0.007	0.002	0.003	0.006	0.006	1.000			
Dim8: candidate’s experience	−0.009	0.005	−0.012	−0.003	0.008	0.009	0.002	1.000		
Vignette order within the deck	−0.002	0.011	−0.011	−0.001	0.003	0.010	0.014	0.021	1.000	
Deck number	0.042	0.002	0.000	0.000	0.000	−0.008	−0.001	−0.022	0.000	1.000
N = 15,036										

### Choice of occupations and vignette auxiliary information

The selection of occupational groups for the FSE evaluation was guided by this study’s objectives. As this research project focused on organisational-level determinants of hiring discrimination, occupations were selected to ensure a high level of organisational diversity. This explains the inclusion of ‘common’ jobs that are found across all industries and are independent of company size or ownership structure.

To identify ethnic discrimination in recruitment, the study focused on medium-skilled occupations where discrimination is likelier to occur than in low-skilled occupations. The latter are often so-called ‘bottleneck jobs’, where discrimination is less likely due to the scarcity of candidates^31^. As gender discrimination was an important dimension of the study, the selected occupations spanned strongly female-dominated occupations, strongly male-dominated occupations and occupations with a more balanced gender composition. Exclusively non-licensed occupations were selected to eliminate the influence of regulations on the evaluation results.

Finally, five jobs were selected for evaluation: office clerk, secretary, ICT technician, bookkeeping clerk and sales worker. Respondents were asked to choose occupation(s) in which they felt competent in evaluating candidates. If multiple occupations were selected, the system assigned the one chosen least often by other respondents to ensure a uniform distribution of occupations. Table 3 shows the occupations as they were presented to the respondents in four language versions.

**Table 3:** Definitions of occupational groups as presented in the master version (English) of the survey and their translations in four language versions.

English	Norwegian	German	Polish	Romanian
Office clerk	Kontormedarbeider	Bürokräfte	Pracownik obsługi biurowej	Funcționar/ă
Secretary	Sekretær	Sekretariatskräfte	Sekretarz/Sekretarka	Secretar/ă
ICT operations and user support technician (e.g. IT consultant, computer network specialist, IT-support specialist)	IKT operatører og brukerstøtte (f.eks. IT-konsulent, nettverks- og systemspesialist, driftseller datatekniker, IT-support)	Techniker/in für den Betrieb von Informations- und Kommunikationstechnologie und für die Anwenderbe-treuung (z. B. IT-Berater/in, Netzwerkspezialist/in, ICT-Techniker/in, IT-Support-Spezialist/in)	Technik informatyk (np. konsultant IT, specjalista ds. sieci komputerowych, technik komputerowy, specjalista ds. wsparcia IT)	Operațiuni TIC și tehnician care oferă asistență pentru utilizatori (de exemplu, consultant IT, specialist în rețea de calculatoare, tehnician în calculatoare, specialist/ă în asistență IT)
Sales worker (e.g. salesperson, shopkeeper, ticket clerk, call centre salesperson)	Salgsmedarbeider (f.eks. selger, butikkeier, billettselger, telefon- og nettselgere)	Verkaufskräfte in Handelsgeschäften (z. B. Verkäufer/in, Ticketverkäufer/in, Callcenter-Verkäufer/in)	Sprzedawca (np. ekspedient, kasjer wydający bilety, sprzedawca w centrum sprzedaży telefonicznej)	Lucrător în vânzări/comercial (de exemplu, vânzător, agent de vânzare, funcționar emitent de bilete, agent de vânzări în call centre)
Accounting and bookkeeping clerks (e.g. assistant bookkeeper, accounting clerk, invoicing clerk)	Regnskapsmedarbeider (f.eks. regnskapsassistent, faktureringsmedarbeider)	Sachbearbeiter/in Buchhaltung (oder auch Buchhaltungsassistent/in, Fakturist/in)	Pracownik do spraw rachunkowości i księgowości (np. fakturzystka, asystent do spraw księgowości, technik rachunkowości)	Funcționar(ă) în servicii de evidență contabilă (de exemplu contabilitate primară, operator facturare)

The auxiliary information – allowing for more precise estimation of main effects – was either identical in all vignettes (all candidates were assumed to have a residence and work permit, as well as two years of work experience in the host country; it was explicitly stated that the employer had received a sufficient number of applications, and that the job position in question was full-time) or linked to specific occupations or countries or nationalities (candidates were assumed to have a typical educational background and command of English for a specific occupation in a specific country; age was fixed at the typical educational level plus two years). Work experience was set at two years in the host country for two reasons: 1) to increase the realism of the candidates presented in the vignettes, which were assumed to have a very good command of the host-country language (B2 or C2) and 2) to signal to respondents that the candidates were already established in the host country and its labour market. Tables 4, 5 show how the auxiliary information was presented to the respondents.

**Table 4:** List of additional information presented in the vignettes that was linked to the job for which a candidate applied.

Country/job varying categories	Germany	Norway	Poland	Romania
**A. Job**	1. ICT technician 2. Office clerk 3. Secretary 4. Bookkeeping/accounting clerk 5. Sales worker			
**B. Age**	1. (25) 2. (22) 3. (22) 4. (22) 5. (22)	1. (25) 2. (25) 3. (25) 4. (25) 5. (22)	1. (22) 2. (25) 3. (25) 4. (25) 5. (22)	1. (25) 2. (22) 3. (22) 4. (25) 5. (22)
**C. Country-job specific educational background**	1. BA in IT (BA-Abschluss im IT-Bereich) 2. Upper secondary, vocational (Ausbildung zum/zur Kaufmann/frau) 3. Upper secondary, vocational (Ausbildung zum/zur Industriekaufmann/frau) 4. Upper secondary, vocational (Ausbildung zum/zur Kaufmann/frau für Büromanagement) 5. Upper secondary, vocational (Ausbildung zum/zur Verkäufer/in)	1. BA in IT (bachelorgrad i IT) 2. BA in Business & Administration (bachelorgrad i administrasjon og ledelse) 3. BA in Business & Administration (bachelorgrad i administrasjon og ledelse) 4. BA in Finance & Accounting (bachelorgrad i økonomi og administrasjon) 5. Upper secondary (videregående utdanning)	1. Upper secondary, vocational (technikum informatyczne) 2. BA in Business & Administration (licencjat na kierunku biznes i administracja) 3. BA in Business & Administration (licencjat na kierunku biznes i administracja) 4. BA in Finance & Accounting (licencjat na kierunku finanse i rachunkowość) 5. Upper secondary, vocational (technikum handlowe)	1. BA in IT (licență în domeniul IT) 2. Upper secondary (liceu) 3. Upper secondary (liceu) 4. BA in Economics (licență in domeniul științe economice) 5. Upper secondary (liceu)
**D. Command of English**	1. ICT technician **(upper-intermediate level (B2))** 2. Office clerk **(intermediate level (B1)) (RO = A2)** 3. Secretary (general) **(intermediate level (B1))** 4. Bookkeeping clerk **(intermediate level (B1))** 5. Sales worker **(elementary level (A2)) (NO = B1)**			

Note: Fixed auxiliary information provided in the vignettes was linked to ‘A. Job’ according to Arabic numbers within a given country (e.g. all candidates applying for the job of ICT technician in Germany were 25 years old, had a Bachelor’s degree in IT and had an upper-intermediate level of English (B2)).

**Table 5:** List of additional information presented in the vignettes linked to the nationality of candidates.

Country/nationality varying categories	Germany	Norway	Poland	Romania
**A. Nationality**	1. German 2. Ukrainian 3. Syrian	1. Norwegian 2. Ukrainian 3. Syrian	1. Polish 2. Ukrainian 3. Belarusian	1. Romanian 2. Ukrainian 3. Nepali
**B. The country where work experience was gained**	1. Germany 2. Germany 3. Germany	1. Norway 2. Norway 3. Norway	1. Poland 2. Poland 3. Poland	1. Romania 2. Romania 3. Romania
**C. Mother tongue**	1. German 2. Ukrainian 3. Arabic	1. Norwegian 2. Ukrainian 3. Arabic	1. Polish 2. Ukrainian 3. Belarusian	1. Romanian 2. Ukrainian 3. Nepali
**D. Host-country language**	1. --- 2. German 3. German	1. --- 2. Norwegian 3. Norwegian	1. --- 2. Polish 3. Polish	1. --- 2. Romanian 3. Romanian

Note: Information is linked to ‘A. Nationality’ according to the Arabic numbers within a given country (e.g. all Syrian candidates applying for any job in Norway: gained professional experience in Norway, had Arabic mother tongue and had a defined level of command of Norwegian).

### Response scales

The candidates presented in the vignettes were evaluated in two ways. First, they were assessed on a typical scale used in the FSEs for hiring decisions. The respondents were asked two evaluation questions:

- How likely is it that you will invite this person for the interview, given the needs and characteristics of your organisation? (0 – very unlikely; 10 – very likely) (variable *d11*)
- How likely is this person to be employed, given the needs and characteristics of your organisation? (0 – very unlikely; 10 – very likely) (*d12*)

Each respondent was presented with six random vignettes (for more information about allocation of vignettes, see “Vignette dimensions and design” in the “Methods” section). There were two reasons for asking the two evaluation questions. The first was practical, as each of the two evaluation methods had its own limitations. Asking about interview invitations may have resulted in less variation and skewed results toward higher scores, as inviting a candidate to an interview carries lower stakes than hiring them. Questions about hiring were more resistant to these issues but involved greater measurement error, since respondents could more accurately assess interview likelihood than hiring likelihood. Hiring involves a more complex process and is harder to evaluate.

**Fig. 1:**
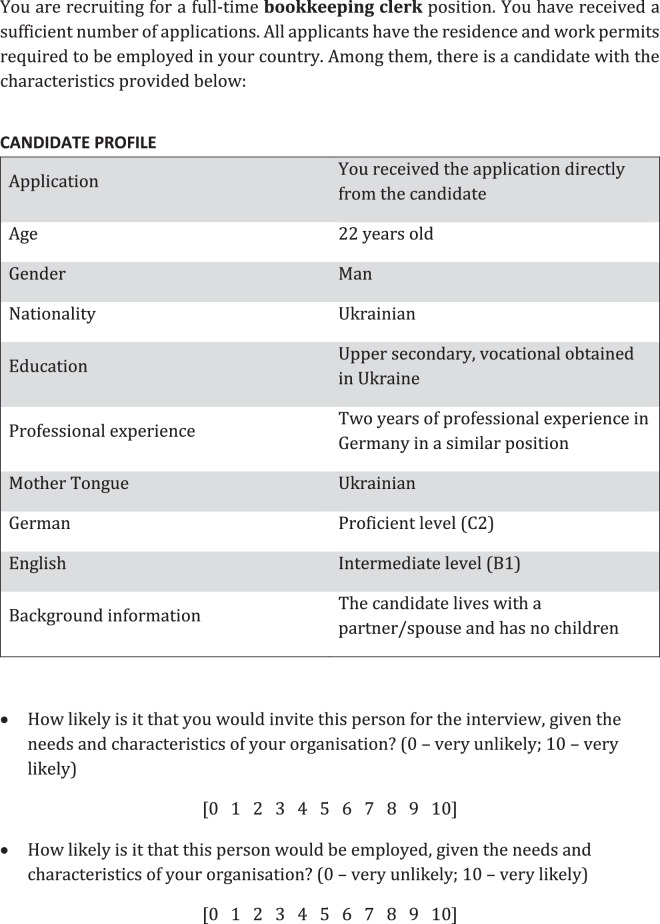
Example of a vignette used in the survey (Germany, English translation).

The second reason was methodological. Existing FSE studies on hiring have used these two response scales interchangeably. Including both evaluation questions in a single survey allows for a systematic comparison of the variability and reliability of these response scales. The order of the response scales varied randomly across respondents and was indicated in variable *d1_order*. The example vignette with the *d11* and *d12* response scales is shown in Fig. 1.

The second type of rating required the respondents to conduct a pairwise comparison of the two highest-scoring hypothetical candidates (based on *d11* scores) using a ranking system:

- If you had to choose one of the two candidates presented below, which candidate would you prefer for the position of (chosen occupation), given the needs and characteristics of your organisation? (*d2*)

In cases where the top-scoring candidates were rated equally, the best candidates were randomly selected from among the highest scorers. This forced choice task should not be confused with a discrete choice experiment^32^, as respondents were asked to make only one choice and the job candidates’ characteristics were not randomly assigned; rather, they represented the most desirable characteristics of candidates. Therefore, the choice task itself does not guarantee orthogonality of dimensions or balance of levels. It should be treated as a version of the FSE in which respondents are forced to choose the best candidate (whereas on rating scales, multiple candidates can be evaluated equally).

### Survey questionnaire

The FSE was integrated into a questionnaire to collect information about the respondents’ characteristics and their organisations. They were asked to describe the organisations for which they recruited, whether as employees or as external recruiters. If they recruited for multiple organisations, they were asked to choose the one they knew best and refer to it throughout the survey. In most questions, respondents were asked to refer not only to their specific organisation but also to the job within that organisation for which the hypothetical candidates in the vignettes applied. In the questionnaire, the respective survey questions are indicated in brackets. Both the questionnaire and the codebook are available in the same repository as the survey dataset.

- Recruitment channels (Q3)
- Information sources used to assess candidates (Q4)
- Persons involved in the recruitment process (Q5)
- Formalisation of the recruitment process (Q7–Q9)
- Opportunities for professional development (Q10)
- Level of interaction with co-workers and customers in the given position (Q11, Q12)
- Flexible working arrangements (Q13)
- Level of job demands related to the concept of greedy jobs^33^ (Q14)
- Diversity-oriented aims and the presence of a diversity manager (Q19–Q21)
- Diversity policy measures applied in the organisation (Q22, Q23)
- Gender and ethnic composition of the organisation (Q26, Q27)
- Other organisational characteristics, including sector of economic activity (Q15), ownership type (Q16), size of the city in which the company is located (Q17), presence of a human resources (HR) department (Q18), company size (Q24) and whether the organisation is national or international (Q25)

The organisational features are complemented by the following respondent characteristics:

- Role in the recruitment process (Q6)
- Whether HR management is the respondent’s main field of professional activity (Q28)
- Experience in recruitment (Q29)
- Level of education and its relevance to HR management (Q30, Q31)
- Sociodemographic characteristics, including age (Q32), gender (Q33), parental status (Q34) and ethnic/national identity (Q35)
- A five-item measure of socially desirable responding (Q36)

To control for socially desirable responding (SDR), which might have biased the estimates—particularly those related to evaluating the candidates–we included in the survey a five-item measure of SDR^34^ (Q36). This measure presented respondents with descriptions of highly desirable behaviours that are rare and undesirable behaviours that are common. Respondents who claimed they engaged in the former (e.g. ‘are always good listeners, no matter whom they are talking to’) or not in the latter (e.g. ‘sometimes feel resentful when they do not get their way’) were considered prone to SDR. In the original article, where the scale was introduced, only extreme responses (‘definitely true’ or ‘definitely false’) were considered indicative of SDR.

### Sampling

The data collection, preceded by the pilot study, took place in the four countries between November 2024 and March 2025. This study targeted individuals with direct experience in employee recruitment, such as managers, business owners, external recruitment professionals and HR specialists. Because the population was very specific and the planned sample size was large, the procedure employed a non-probability sampling approach. The online survey was implemented by GfK Polonia, which used subcontracted respondent panels.

Where possible, efforts were made to enlarge the sample. This was done in Germany, where additional data were collected using the email addresses of managers and recruiters obtained from the consulting company Dun & Bradstreet (D&B) and from the federal HR managers’ association (Bundesverband der Personalmanager*innen, BMP). Ultimately, the following sources were used by the survey provider, focusing mainly on well-established respondent panels: Cint, Daisycon, Dynata, Norstat and Talk Online Panel. Due to the agreement between GfK Polonia and the data providers, their names were anonymised (single capital letters). Data acquired through email invitations (BPM, D&B) have been indicated in the dataset as ‘own’.

Particular attention was given to selecting appropriate respondents. Therefore, two levels of respondent screening were employed. First, a pre-selection within the respondent panels targeted adults currently working in recruitment-related roles in organisations with 10 or more employees. The incidence rates (i.e., the ratio of those satisfying the eligibility criteria for survey participation to all panel members) amounted, on average, to 9.9 percent in Romania, 10.9 percent in Germany, 14.9 percent in Norway, and 15.9 percent in Poland. Second, the survey applied four additional screening questions: (1) respondents completed the attention-check question by naming the animal shown in a picture; (2) respondents had to select ‘Hiring of employees’ in a multiple-choice question about the main fields of their professional responsibilities (see Q0 in the questionnaire); (3) they were required to refer to a specific organisation they recently recruited for and felt able to describe (see Q1); and (4) they needed to select at least one of the five occupations for which they felt qualified to assess candidates (see Q2 in the questionnaire). Respondents who failed to meet any of these criteria were excluded.

## Data Records

The dataset associated with this article is stored on OSF^35^ repository at 10.17605/OSF.IO/74Q69 in two data formats (STATA hiring_discrimination_dataset.dta; MS Excel hiring_discrimination_dataset.xlsx) and is accompanied by the survey questionnaire and the codebook describing each variable. The data are provided in long format: each column represents a variable, and each row corresponds to the evaluated vignette—a hypothetical job candidate whose characteristics are indicated by variables with the prefixes *x1–x8*, and whose ratings are captured by variables with the prefix *d*. Vignettes are nested within respondents identified by unique IDs. Each respondent evaluated six vignettes, and their display order was recorded in the variable *vorder*. The survey variables’ names correspond to those indicated in the questionnaire and the codebook. The dataset includes a set of auxiliary variables that are recommended to be used as additional exclusion criteria (see “Technical Validation” section).

The dataset does not distinguish between respondent and organisation levels, as the anonymous data collection procedures did not allow for the identification of cases where multiple respondents came from the same organisation. All the variables’ values are presented in English, and information on the country of data collection is contained in the variable *country*. As a result, the dataset has a three-level structure: vignettes nested within respondents nested within countries. Additionally, the variable *source* indicates the anonymised respondent panel from which the data were obtained—some of which, in the case of international providers, span multiple countries.

## Technical Validation

### Pilot study

The data collection phase was preceded by the soft launch of the survey, which was conducted on a sample of 546 respondents (3276 vignettes) in all four countries. During these pilots, we verified the following aspects:

- Question quality and respondent comprehension were assessed through open-ended responses and patterns of missing data.
- Overall survey quality was evaluated via open-ended responses and questionnaire completion times.
- Distributions of key variables used to inform potential recoding and refinement of selected categories.
- Survey logic and technical functioning, including branching, filtering and the alignment of numeric dataset codes with the values shown in the survey.
- Algorithms’ performance, such as uniform assignment of occupations, random vignette allocation and selection of top-rated vignettes (see variable *d2)*.

The soft launch was complemented by several structured interviews with recruiters to assess the external validity of the vignettes—specifically, whether they provided sufficient information for candidate evaluation and whether such information was typically available at the early stage of recruitment.

### Data quality assessment

The main phase of data collection adopted a multidimensional data quality assessment system, taking into account the criteria listed below:

- **Duplicate Prevention:** The system uses digital fingerprinting to identify and reject duplicate responses submitted from the same computer, ensuring that each respondent participates only once in a survey.
- **IP not survey country; IP not survey or border country**: The IP address of the respondent does not match the country (or bordering country) in which the survey is supposed to be conducted.
- **Browser not survey language (excl. English**): The browser language does not match the interview language.
- **High outlier:** too many options chosen (>5) in a screening question (about the main fields of professional responsibilities, see Q0 in the questionnaire).
- **Screen size 30%: >**30% of respondents within a panel with 10 + respondents have the same screen size (to detect click-farm that uses the same device or type of device to fill in many surveys via one (sub) panel).
- **Speeder > = 30%:** > = 30% of the questions that a respondent answered are flagged for speeding (i.e. answered in < = 50% of the overall median duration).
- **Speeder > = 50%:** > = 50% of the questions that a respondent answered are flagged for speeding (i.e. answered in < = 50% of the overall median duration).
- **Slow-poker > = 20%:** > = 20% of short questions are ‘slowpoked’ (i.e. questions with a median duration of < = 5 seconds are answered in >250% of the median duration).
- **Suspicious panel:** Data come from the panel containing >60% respondents, with at least one flag or >30% respondents with at least three flags.

If a respondent met an exclusion criterion, his or her response was flagged. Receiving two or more flags resulted in the respondent’s data being removed from the database. Furthermore, respondent panels with a high number of flagged cases were also rejected (see the “suspicious panel” criterion). Table 6 presents the number of rejected respondents broken down by the exclusion criterion (type of flag).

**Table 6:** Overview of data quality control criteria.

Count	IP not survey country	IP not in the survey or border country	Browser not survey language (excl. English)	High outlier	Screen size 30%	Slow-poker > = 20%	Speeder > = 30%	Speeder > = 50%	Suspicious panel
384						x			
167							x		
120						x			x
109							x	x	
93									x
67					x				x
53					x				
38					x		x		x
36				x					
28			x						
23					x	x			x
18			x			x			
12							x		x
11					x		x	x	x
9					x	x			
8						x	x		x
7	x	x							
5			x				x		
5					x	x	x		x
3	x	x					x		
2	x								
2	x	x				x			
2	x		x						
2	x					x			
2			x			x	x		
2			x				x	x	
2				x		x			
2						x	x		
2				x			x		
1	x	x	x			x			
1	x		x			x			
1	x				x	x			
1			x	x					
1			x		x				
1	x	x			x		x	x	
1	x		x				x	x	
1					x		x	x	
1						x	x	x	
1				x			x	x	
1							x	x	x
1	x	x			x		x		x
1	x	x				x			x
1	x	x		x		x			x

Table 6  also includes those who received only one flag and were, therefore, not excluded. Finally, 558 respondents were excluded, resulting in a final sample of 2,506 respondents who evaluated 15,036 vignettes.

Additionally, the dataset includes two variables measuring the completion time of vignettes and the entire survey (in seconds). In most previous applications, we used a criterion requiring that the first or second vignette be evaluated in at least 15 seconds—the estimated minimum time needed to read the instructions and the vignette with understanding. Another strategy is to exclude cases with a total response time less than 50% of the median. We recommend applying additional exclusion criteria, although this is left to the users’ discretion. The variables that can be used for this purpose are presented in Table 7.

**Table 7:** Variables to be used for additional exclusion criteria.

Variable Name	Description	Measurement
*total_time*	Total time to complete the questionnaire (in seconds)	scale
*vign_time*	Time to complete the vignette (in seconds)	scale
*vorder*	Order of the vignette as displayed to a respondent (can be used in combination with *vign_time*)	ordinal
*sum_of_flags*	Number of flags the respondent received (0 or 1)	nominal
*flag_comment*	Description of the type of flag the respondent received (if any)	string

## Data Availability

The dataset, the codebook and the survey questionnaire are available at OSF^35^ repository 10.17605/OSF.IO/74Q69.

## Code availability

No additional code accompanies the dataset.
